# Ozone Induces Distress Behaviors in Fig Wasps with a Reduced Chance of Recovery

**DOI:** 10.3390/insects12110995

**Published:** 2021-11-05

**Authors:** Maryse Vanderplanck, Benoit Lapeyre, Shéhérazade Lucas, Magali Proffit

**Affiliations:** 1 Centre d’Écologie Fonctionnelle et Évolutive (CEFE), Université de Montpellier, CNRS, EPHE, IRD, 34293 Montpellier, France; benoit.lapeyre@cefe.cnrs.fr (B.L.); magali.proffit@cefe.cnrs.fr (M.P.); 2 Institut d’Urbanisme et de Géographie Alpines (IUGA), Université Grenoble Alpes, 38000 Grenoble, France; sheherazade.lucas@outlook.com

**Keywords:** Agaonidae, air pollution, abiotic risk, pollinator, behavioral change, recovery

## Abstract

**Simple Summary:**

Ecological interactions among organisms underpin the stability of ecological networks, which are responsible for species biodiversity in ecosystems. These interactions are currently threatened by environmental risks, mainly due to human activities, such as air pollution. Among air pollutants, tropospheric ozone (O_3_) is known to disrupt chemical communication between plants and their pollinators. Alarmingly, its concentration is likely to increase by two–four-fold in the next two decades. However, the direct effects of O_3_ on the behavior of pollinators themselves have not been investigated so far, even though insect behavior is key to their ecological interactions. In this study, we evaluated the potential effects of O_3_ at different field-realistic concentrations on the behavior of the fig wasp *Blastophaga psenes*, the exclusive pollinator of the Mediterranean fig species *Ficus carica.* We found that O_3_, even at low concentrations, induced abnormal motility in fig wasps, and that exposed individuals might only have a reduced chance of recovery. Overall, our findings indicate that O_3_ can affect pollinator behavior, which may have detrimental implications for pollination systems.

**Abstract:**

Among anthropogenic environmental risks, air pollution has the potential to impact animal and plant physiology, as well as their interactions and the long-term survival of populations, which could threaten the functioning of ecosystems. What is especially alarming is that the concentration of tropospheric ozone (O_3_) has dramatically increased since pre-industrial times. However, the direct effects of O_3_ on the behavior of pollinators themselves have not been investigated so far even though insect behavior is key to their ecological interactions, which underpin the stability of ecological networks responsible for species biodiversity in ecosystems. In this study, we aim to determine the potential effects of O_3_ episodes at different field-realistic concentrations (0, 40, 80, 120, and 200 ppb for 60 min) on the behavior of the fig wasp *Blastophaga psenes* by monitoring exposed individuals hourly for 5 h after exposure. We found that ozone episodes induced major changes in insect behavior, which were already significant at 80 ppb with individuals displaying abnormal motility. The tracking over time clearly showed that exposed individuals might only have a reduced chance of recovery, with a decreasing proportion of active fig wasps despite the cessation of an O_3_ episode. These findings illustrate that O_3_ episodes can affect pollinator behavior, which may have detrimental implications for pollination systems. It is, therefore, of importance to assess the effects of O_3_ on insect behavior in order to predict how it could modify ecological interactions and species biodiversity in ecosystems.

## 1. Introduction

Current global change substantially threatens the balance of ecosystems through decreased species abundance and diversity [1]. Many synergistic drivers contribute to this loss in biodiversity, including habitat destruction/fragmentation [2,3], the use of agrochemicals [4,5], decreasing resource diversity [6], climate change [7,8], and air pollution [9,10]. All these environmental risks impact living organisms through changes in their phenology, distribution, and behavior [11,12]. Such processes depend on species’ ecological traits and often involve cascading effects [13]. Among these effects, changes in behavior may lead to drastic modifications and/or disruptions of crucial species interactions, including plant–pollinator interactions [11].

Among pollinators, insects exhibit spectacular and diverse behaviors that are key to their participation in ecological interactions. These behaviors can be acutely sensitive to environmental stressors, such as exposure to chemicals [14]. Specifically, a change in behavior is very often the first response of an organism to an environmental change. Such behavioral modifications can potentially facilitate adaptation in a changing world by improving the survival and reproduction of species, or hinder it by interfering with physiological processes or disrupting species interactions (reviewed in [11]). These interactions between species underpin the stability of the ecological networks that shape biodiversity. Understanding changes in the behavior of interacting species is therefore essential to predict the future stability of ecological networks in a changing world. However, the effects of environmental stressors on pollinator behaviors remain understudied, especially the effect of air pollution. Tropospheric ozone (O_3_) is one of the most damaging air pollutants, known to have detrimental effects on organisms [15,16], and whose concentration is likely to increase by two–four-fold in the next two decades [17]. Here, we tested whether ozone exposure episodes (local seasonal peaks of >40 ppb O_3_) result in a change in the behavior of the fig wasp *Blastophaga psenes*, the exclusive pollinator of the Mediterranean fig species *Ficus carica*, and discuss the implications for the fig/fig wasp pollination system.

## 2. Materials and Methods

### 2.1. Biological Model

*Blastophaga psenes* (Hymenoptera, Agaonidae) is a solitary and tiny fig wasp, which is diurnal (active during the day), lives generally less than one day, and does not feed at the adult stage [18]. This fig wasp species is intimately associated with *Ficus carica* (Moraceae) for its reproduction and is its exclusive pollinator. *Blatophaga psenes* has two generations per year that coincide with flowering of fig trees. Wasp larvae overwinter as the last larval stage in the male figs produced in summer. In spring, the overwintering wasps complete their development and emerge in summer in search of receptive figs (usually between 10 am and 5 pm), generally located on a different tree, in which they multiply [18,19]. Because of their ecological traits, fig wasps are probably highly susceptible to environmental stressors, as they have a reduced chance of recovery (i.e., reduced lifespan) and plasticity (i.e., reduced lifespan and highly host-specialized interaction), and no possibility for nutritional resilience (i.e., an inability to feed as adults).

### 2.2. Ozone Exposure and Behavioral Assays

**Figure 1:**
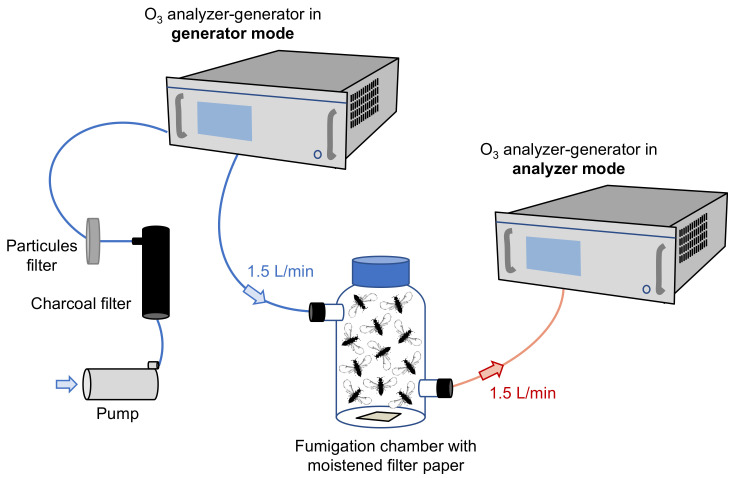
Experimental setup for the controlled and continuous flow during O_3_ exposure of fig wasps at 0, 40, 80, 120, or 200 ppb.

In July 2018, newly emerging adult female wasps were collected from mature figs, taken randomly from different male trees (“Terrain d’Expérience” of the “Centre d’Écologie Fonctionnelle et Évolutive—CEFE”, pesticide-free, Montpellier, France), and tested shortly after their exit from their natal fig. Groups of fig wasps (around 11 individuals) were placed into a laboratory fumigation chamber, which consisted of a glass bottle of 500 mL with a filter paper of 2 cm × 2 cm loaded with 200 μL of distilled water prior to O_3_ exposure. Ozone was produced using the photolysis of molecular oxygen subjected to UV radiation at a wavelength of 185 nm (UV photometric Ozone Analyzer, Model 49i, Thermo Scientific) and delivered continuously in a flow through the fumigation chamber (flow rate of 1.5 L·min^−1^). One extremity of the fumigation chamber was connected to an analyzer generator in the generator mode, pushing air containing different O_3_ concentrations into the bottle at a flow rate of 1.5 L·min^−1^, while the other extremity was connected to an analyzer generator in the analyzer mode, where air was extracted at a flow rate of 1.5 L·min^−1^ to ensure that the desired O_3_ concentration was present in the bottle. Before entering the system, the air was cleaned of any VOCs by using an activated carbon filter. We used exclusively Teflon tubes to connect the pump, the VOC filter, and the O_3_ generator and analyzer (Figure 1). Individuals were exposed to 0 (control, *n* = 10 with 11 ± 3 individuals per trial), 40 *(n* = 10 with 11 ± 2 individuals per trial), 80 (*n* = 11 with 11 ± 3 individuals per trial), 120 (*n* = 9 with 12 ± 1 individuals per trial), or 200 ppb (*n* = 10 with 11 ± 3 individuals per trial) for 60 min based on the recorded O_3_ episodes in the French Mediterranean region [17,20,21]. All O_3_ exposures were conducted in a greenhouse (“Terrain d’Expérience”) maintained at 25 °C under natural light, generally between 11 a.m. and 12 a.m.

After O_3_ exposure, the fumigation chambers were disconnected from the O_3_ generator and analyzer and maintained at 25 °C under natural light inside the greenhouse. We then performed observations to assess the probability of motility of the exposed fig wasps, without disturbing them inside the glass bottle. Groups of individuals were monitored hourly for 5 h to track survival and record behavioral responses. Five distinct behaviors were observed: (1) motility, (2) abnormal motility, (3) moderate distress behavior (i.e., individuals alternately lying on their backs or upright), (4) severe distress behavior (i.e., individuals lying on their backs, unable to right themselves), and (5) death.

### 2.3. Statistical Analyses

All analyses were performed in R version 3.4.0 [22]. To estimate the probability of an individual displaying motility after an O_3_ episode, non-parametric Kaplan–Meier curves were estimated using the “survfit” function (R package “survival”). Individuals that displayed abnormal motility or distress behaviors (including death) were the exact observations, while those that remained active or were not followed-up until the end of the survival tracking were the censored data. We used a log-rank test (“survdiff” function, R package “survival”) to test for differences in Kaplan–Meier curves among the O_3_ concentrations. Post hoc tests were conducted using pairwise log-rank tests adjusted for false discovery rate (FDR) (“pairwise_survdiff” function, R package “survminer”). Furthermore, we used multivariate generalized linear models (ManyGLM) to analyze the effects of O_3_ exposure on fig wasp behavior in detail (“mvabund” R package [23]). By correcting for negative binomial responses, this multivariate method allows for the handling of zero-inflated multiple count data without normality assumption [24,25]. Moreover, it has more statistical power than distance-based multivariate analyses such as perMANOVA [25,26]. As there are no extant mixed-effect models for the ManyGLM method, we analyzed the effect of O_3_ exposure for each hour of survival tracking separately. When significant difference was detected, multiple pairwise comparisons were conducted followed by the univariate test procedure implemented in ManyGLM. We reported unadjusted *p*-values for all tests, because the detectability after adjustment for multiple comparisons would be very low given the high number of comparisons, but interpreted the results with caution [27,28]. 

## 3. Results and Discussion

**Figure 2:**
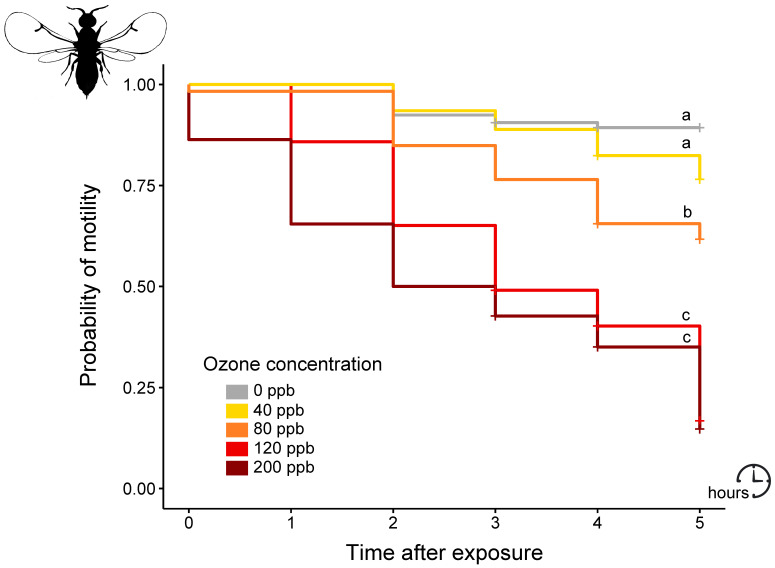
Kaplan–Meier curves depicting the probability of motility over time for each O_3_ regime. Crosses indicate points of censored data. Different letters indicate significant differences (*p* < 0.05) in the probability of motility between O_3_ regimes based on pairwise log-rank tests.

The probability of an individual displaying motility after an O_3_ episode significantly differed according to the O_3_ concentration (Kaplan–Meier analysis, χ^2^ = 180, df = 4, *p* < 0.001). While control individuals (exposed to 0 ppb O_3_) remained mostly active over time and even until the end of the tracking (after five hours), the individuals exposed to O_3_ exhibited a decreasing probability of being active over time, which was significant at 80, 120, and 200 ppb O_3_ (*p* < 0.001 for 0 vs. 80 ppb O_3_, 0 vs. 120 ppb O_3_ and 0 vs. 200 ppb O_3_) with an even more pronounced decrease at 120 and 200 ppb O_3_ compared to 80 ppb O_3_ (*p* < 0.001 for 80 vs. 120 ppb O_3_ and 80 vs. 200 ppb O_3_) (Figure 2, Appendix A).

**Figure 3:**
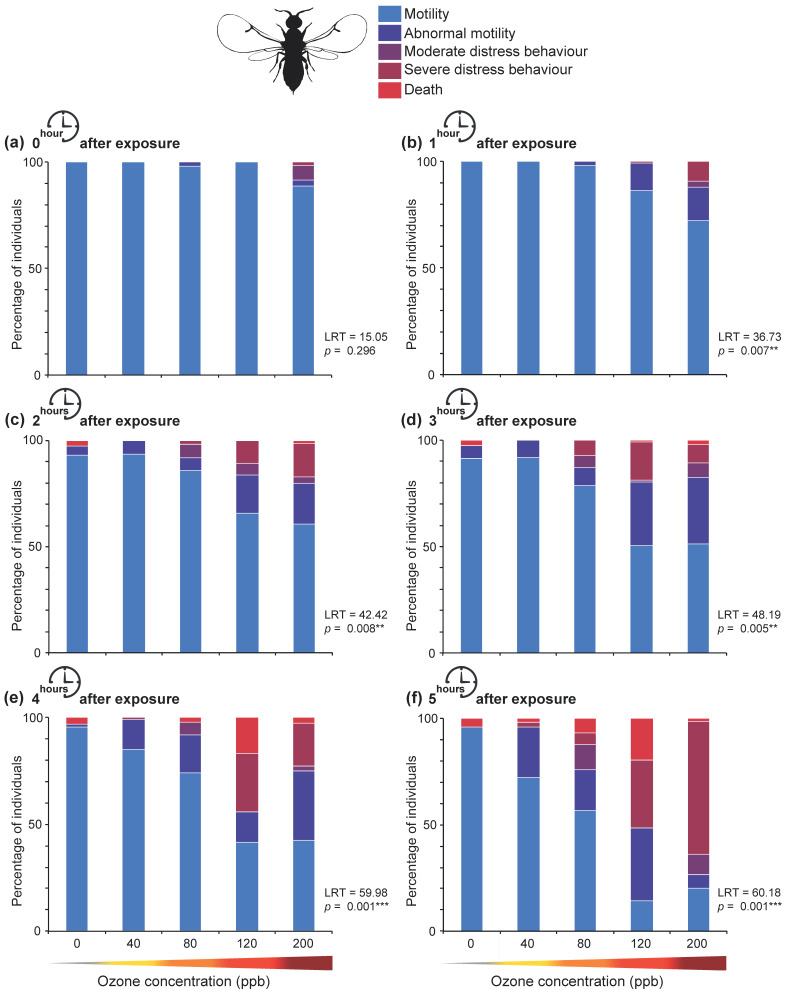
Behavior of fig wasps exposed to O_3_ regimes for one hour (0, 40, 80, 120, and 200 ppb). After O_3_ exposure, individuals were left in climate-controlled conditions and monitored hourly for 5 h to track survival and record behavioral responses. Each subfigure (**a**–**f**) corresponds to one of the monitoring moments. Five distinct behaviors were observed: (1) motility, (2) abnormal motility, (3) moderate distress behavior, (4) severe distress behavior, and (5) death. Data are expressed as the mean percentage of individuals displaying the different behaviors. Asterisks indicate significant differences (**, very significant, *** extremely significant) in the behavior of exposed fig wasps between O_3_ regimes.

The multivariate analyses showed that the proportion of individuals displaying abnormal motility significantly increased one hour after O_3_ exposure at 120 and 200 ppb compared to both the control (0 ppb) and 40 ppb (0 vs. 120 ppb O_3_, *p* = 0.011; 40 vs. 120 ppb O_3_, *p* = 0.016; 0 vs. 200 ppb O_3_, *p =* 0.042; and 40 vs. 200 ppb O_3_, *p* = 0.021) (Figure 3b, Appendix A). At three hours after the O_3_ episode the proportion of individuals displaying motility significantly decreased when exposed to 200 ppb O_3_ compared to the control (*p* = 0.003), 40 (*p* = 0.001), and 80 ppb O_3_ (*p* = 0.037), while individuals exposed to 120 ppb O_3_ showed moderate distress behavior more frequently compared to both the control (*p* = 0.036) and 40 ppb O_3_ (*p* = 0.011) (Figure 3d, Appendix A). At four and five hours after the O_3_ episode the effects became significant for individuals exposed to 40 and 80 ppb O_3_ (i.e., frequent abnormal motility) (four hours after exposure: 0 vs. 40 ppb O_3_, *p* = 0.024; 0 vs. 80 ppb O_3_, *p* = 0.032; five hours after exposure: 0 vs. 40 ppb O_3_, *p* = 0.008; 0 vs. 80 ppb O_3_, *p* = 0.04), while effects were even stronger for individuals exposed to 120 and 200 ppb O_3_, with only a low proportion displaying motility and an increase in the number of individuals displaying severe distress behaviors (four hours after exposure: 0 vs. 120 ppb O_3_, *p* = 0.019; 0 vs. 200 ppb O_3_, *p* = 0.001; five hours after exposure: 0 vs. 120 ppb O_3_, *p* = 0.003; 0 vs. 200 ppb O_3_, *p* = 0.004) (Figure 3e,f, Appendix A).

Such negative effects on fig wasps’ behavior could be explained as due to oxidative stress triggered by O_3_ episodes. Previously, O_3_ has been shown to induce molecular damage such as the oxidation of proteins, lipid peroxidation, and damage to DNA; it also causes the deregulation of intracellular signal transduction, which could disrupt the whole organism and lead to death (reviewed in [29]). To support this hypothesis, further experiments are required to evaluate the direct effect of O_3_ on fig wasp individuals by assessing oxidative stress biomarkers, such as levels of lipid peroxidation and total reactive antioxidant potential [29,30]. However, as fig wasps probably vary in their susceptibility to O_3_ (i.e., among-individual variation), differences in these oxidative stress markers should be assessed in several individuals every hour during the hourly monitoring, which involves an experimental setup with adequate replicates. Indeed, comparing the effect of exposure regimes by assessing oxidative stress markers only in surviving individuals at the end of the trials (i.e., after five hours) would lead to biases, such as the absence of difference, as only the tolerant fig wasps would be accounted for (i.e., oxidative stress underestimated) [30]. With respect to the chances of recovery of O_3_-exposed fig wasps, our study noted that the effect of exposure lasted up to five hours after the cessation of O_3_ exposure, at least, with even stronger effects over time. This limited possibility of recovery is reinforced by the ecological traits of this pollinator species. Indeed, the flight period of fig wasps (i.e., diurnal species, active between 10 a.m. and 5 p.m.) exposes them to high O_3_ concentrations. As an additional point, their reduced lifespan (generally less than 24 h), as well as their inability to feed as adults, does not allow for the activation of endogenous antioxidant defense systems (i.e., energetically costly processes requiring time to be activated) [31] and prevents them from benefiting from a protective effect of dietary antioxidants [32,33].

By decreasing the proportion of active fig wasps and therefore the proportion of fig wasps that could efficiently encounter their host, O_3_ episodes are expected to disrupt the fig/fig wasp’s pollination system. To support this hypothesis, it would be interesting to test whether the effects of O_3_ on fig wasp motility has implications for their ability to disperse to other trees as well as for their ability to enter, pollinate, and oviposit into receptive figs. Besides these O_3_ effects on pollinator behavior, O_3_ also has significant effects on the detection of volatile organic compounds responsible for pollinator attraction [16]. The reproductive success of both fig wasps and figs might hence be already affected by the current O_3_ episodes in the French Mediterranean region. Indeed, fig wasp females actively search for receptive figs of *F. carica* around midday, which corresponds to the occurrence of daily maximum O_3_ concentrations. The female wasps that emerge from their natal fig during a high O_3_ incidence would display impaired motility and might not be capable of reaching their host plant. Such an impact of O_3_ episodes on pollinators, including the fig wasps, could easily trigger a domino effect by impacting insect–plant interactions, thereby causing a more widespread disruption of pollination networks, threatening ecosystem stability.

## Data Availability

Data available on request from the authors.

## Appendix A

**Table A1:** Behavior of fig wasps exposed to O_3_ regimes for one hour (0, 40, 80, 120, and 200 ppb). After O_3_ exposure, individuals were left in climate-controlled conditions and monitored hourly for 5 h to track survival and record behavioral responses. Five distinct behaviors were observed: (1) motility, (2) abnormal motility, (3) moderate distress behavior (i.e., individuals alternately lying on their backs or upright), (4) severe distress behavior (i.e., individuals lying on their backs, unable to right themselves), and (5) death. Data are expressed as the number of individuals displaying the different behaviors (mean ± sd), per O_3_ regime and over time.

**Exposure at 0 ppb O_3_ (*n* = 10 with 11 ± 3 Individuals per Trial)**						
	0 h	1 h	2 h	3 h	4 h	5 h
Motility	11 ± 3	11 ± 3	10 ± 3	10 ± 3	9 ± 3	9 ± 3
Abnormal motility	0 ± 0	0 ± 0	1 ± 2	1 ± 2	0 ± 0	0 ± 0
Moderate distress behavior	0 ± 0	0 ± 0	0 ± 0	0 ± 0	0 ± 0	0 ± 0
Severe distress behavior	0 ± 0	0 ± 0	0 ± 0	0 ± 0	0 ± 0	0 ± 0
Death	0 ± 0	0 ± 0	0 ± 1	0 ± 1	0 ± 1	0 ± 1
**Exposure at 40 ppb O_3_ (*n* = 10 with 11 ± 2 Individuals per Trial)**						
	0 h	1 h	2 h	3 h	4 h	5 h
Motility	11 ± 2	11 ± 2	10 ± 2	10 ± 2	9 ± 3	8 ± 3
Abnormal motility	0 ± 0	0 ± 0	1 ± 1	1 ± 1	2 ± 2	3 ± 2
Moderate distress behavior	0 ± 0	0 ± 0	0 ± 0	0 ± 0	0 ± 0	0 ± 0
Severe distress behavior	0 ± 0	0 ± 0	0 ± 0	0 ± 0	0 ± 0	0 ± 0
Death	0 ± 0	0 ± 0	0 ± 0	0 ± 0	0 ± 0	0 ± 0
**Exposure at 80 ppb O_3_ (*n* = 11 with 11 ± 3 Individuals per Trial)**						
	0 h	1 h	2 h	3 h	4 h	5 h
Motility	11 ± 3	11 ± 3	9 ± 3	8 ± 3	8 ± 2	7 ± 3
Abnormal motility	0 ± 0	0 ± 0	1 ± 1	1 ± 1	2 ± 2	2 ± 3
Moderate distress behavior	0 ± 0	0 ± 0	1 ± 2	1 ± 1	1 ± 1	1 ± 2
Severe distress behavior	0 ± 0	0 ± 0	0 ± 1	1 ± 3	0 ± 0	1 ± 2
Death	0 ± 0	0 ± 0	0 ± 0	0 ± 0	0 ± 1	1 ± 1
**Exposure at 120 ppb O_3_ (*n* = 9 with 12 ± 1 Individuals per Trial)**						
	0 h	1 h	2 h	3 h	4 h	5 h
Motility	12 ± 1	10 ± 2	8 ± 4	6 ± 5	5 ± 5	2 ± 3
Abnormal motility	0 ± 0	2 ± 2	2 ± 3	4 ± 4	2 ± 2	4 ± 5
Moderate distress behavior	0 ± 0	0 ± 0	1 ± 1	0 ± 0	0 ± 0	0 ± 0
Severe distress behavior	0 ± 0	0 ± 0	1 ± 2	2 ± 4	3 ± 5	4 ± 5
Death	0 ± 0	0 ± 0	0 ± 0	0 ± 0	2 ± 5	3 ± 6
**Exposure at 200 ppb O_3_ (*n* = 10 with 11 ± 3 Individuals per Trial)**						
	0 h	1 h	2 h	3 h	4 h	5 h
Motility	9 ± 4	7 ± 5	6 ± 3	5 ± 3	4 ± 2	2 ± 2
Abnormal motility	0 ± 1	2 ± 4	3 ± 4	4 ± 4	4 ± 4	1 ± 1
Moderate distress behavior	1 ± 3	0 ± 1	0 ± 1	1 ± 3	0 ± 1	2 ± 4
Severe distress behavior	0 ± 1	1 ± 3	2 ± 4	1 ± 4	3 ± 5	7 ± 4
Death	0 ± 0	0 ± 0	0 ± 0	0 ± 0	0 ± 0	0 ± 0
